# Post-traumatic Left Ventricular Apical Aneurysm With Endoluminal Thrombus

**DOI:** 10.7759/cureus.32348

**Published:** 2022-12-09

**Authors:** George Korelidis, Rory McFadyen, Ghaith Qsous, Samantha Richmond, Renzo Pessotto

**Affiliations:** 1 Cardiothoracic Surgery, Royal Infirmary of Edinburgh, Edinburgh, GBR; 2 Cardiothoracic Surgery, University of Aberdeen, Aberdeen, GBR

**Keywords:** myocardial infarction, arrhythmia, cardiac surgery, aneurysmatectomy, left ventricle

## Abstract

Here, we report a case of a 54-year-old man who presented with complications of a left ventricle apical aneurysm, which was the result of a myocardial infarction (MI). Interestingly, he sustained the MI following a cardiac contusion whilst playing rugby 32 years ago. He had another MI 10 years later, despite the presence of normal coronary angiography following the initial event, and presented with two episodes of sustained ventricular tachycardia over six months. The patient proceeded to surgical resection of the aneurysm and went on to make a good recovery.

## Introduction

A true left ventricular aneurysm (LVA) is a dyskinetic bulging of the LV wall and is usually filled with thrombus. LVAs are lined with a thin wall of scar tissue, which replaces the muscle that undergoes necrosis following an index ischaemic event. In contrast, a false aneurysm appears following the contained rupture of the ventricle [1]. An LVA is one of the most common post-infarction complications occurring in 10% to 35% of patients with MI [1]. It is most frequently found in those with acute anterior transmural MI but may also develop as a result of cardiac trauma, Chagas disease, infective endocarditis, sarcoidosis, or secondary to mid-ventricular hypertrophic obstructive cardiomyopathy (MOHCM), which is associated with LV apical aneurysm [2]. The development of LVAs following myocarditis is also described in the literature [3-6]. The majority of LVAs are formed on the apical and anteroseptal walls, which may be explained by the anatomy of the thinner myocardial wall at the apex (three layers of muscle at the apex compared with four layers at the base). Most LVAs are asymptomatic and are noted during routine tests. However, LVAs can manifest as thromboembolic events, potentially lethal ventricular tachyarrhythmias, heart failure symptoms, recurrent infarction and even sudden cardiac death secondary to rupture [4]. Almost one-third of patients with a ventricular aneurysm develop arrhythmias; the location of arrhythmogenic focus is usually found at the junction of the normal and affected myocardium. This is associated with poor prognosis at presentation and significantly reduced both short-term and long-term survival [1].

## Case presentation

**Figure 1:**
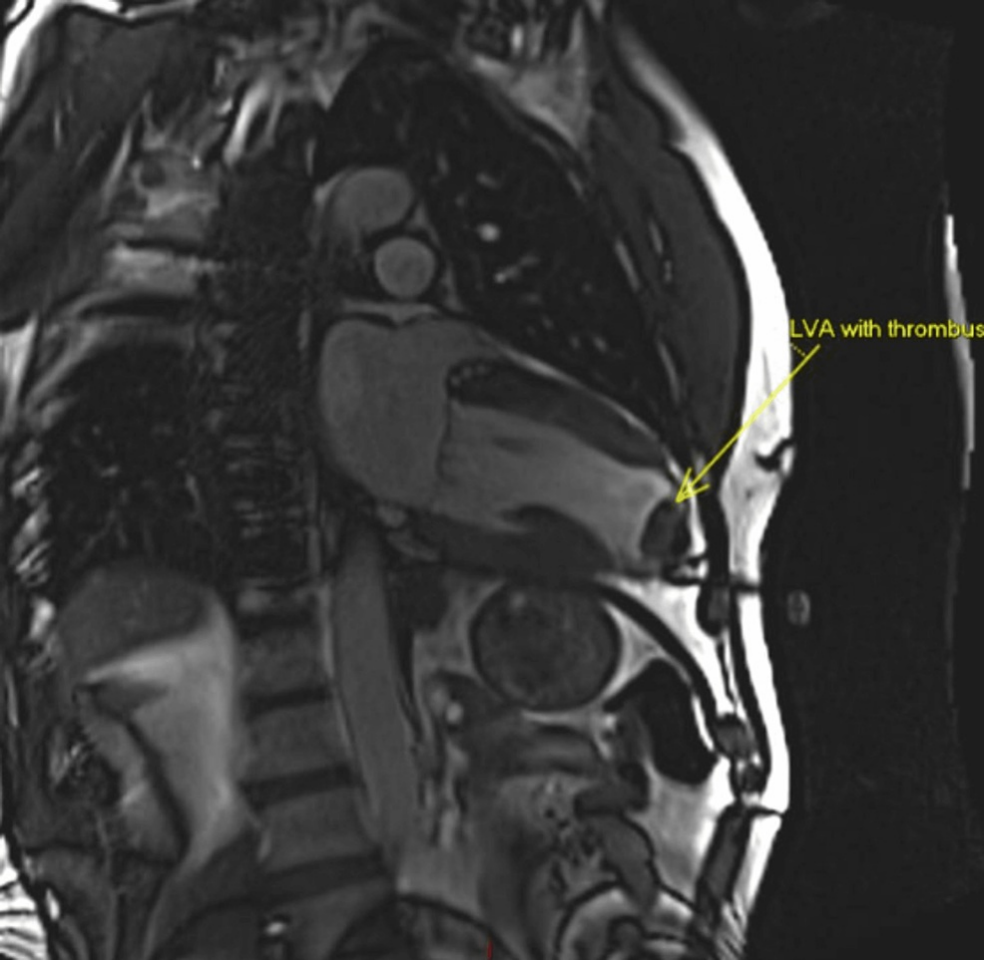
LV apical aneurysm with thrombus LV: left ventricular

The patient was a 54-year-old man with a past history of anterior MI following a cardiac contusion whilst playing rugby 32 years prior. This was treated at the time with tissue plasminogen activator (tPA) thrombolysis and his predischarge tests (ECG, blood and echocardiogram) were satisfactory. A subsequent coronary angiogram was entirely normal. The patient then re-presented with a raised troponin and non-ST-elevation myocardial infarction (NSTEMI) 10 years later. His coronary angiogram was again normal and an echocardiogram showed preserved left ventricular (LV) systolic function, but with a hypokinetic basal septum and apex, and a thinned wall. Meanwhile, MRI demonstrated mild LV impairment and a thinned akinetic filling defect at the apex of the left ventricle consistent with old resolving apical thrombus (Figure 1) and previous MI. Following this second MI, the patient was commenced on warfarin and remained anticoagulated until his eventual surgery.

**Figure 2:**
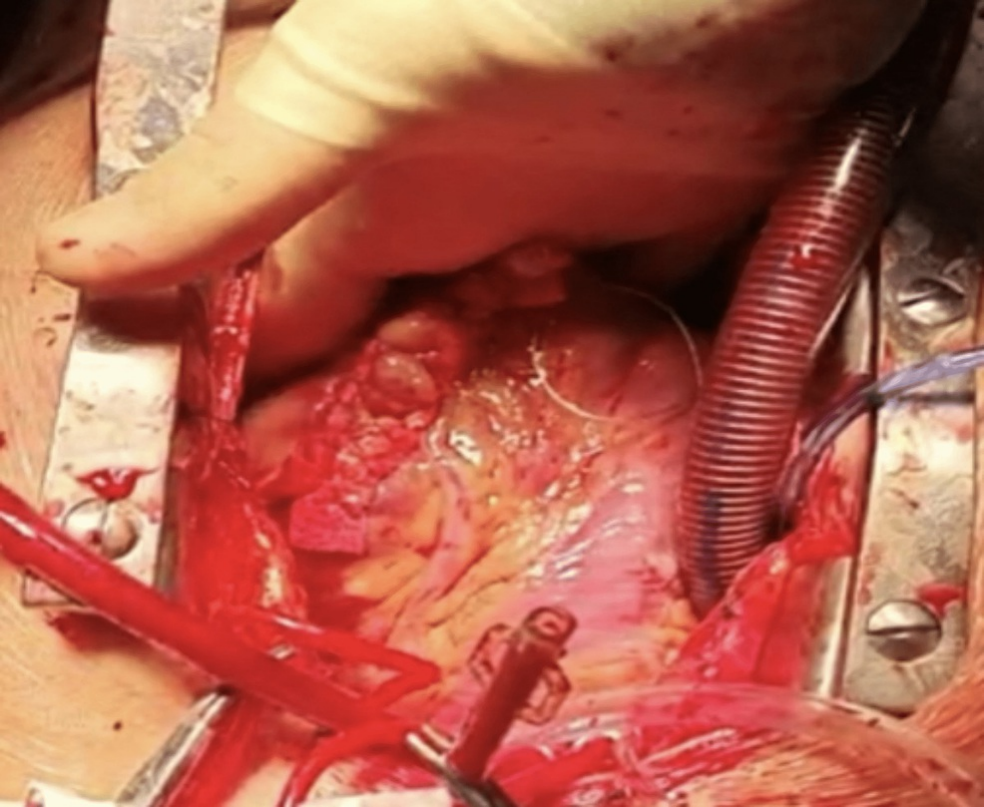
Appearance of LV post linear aneurysmectomy repair LV: left ventricular

Over the next six months, he reported multiple episodes of ventricular tachycardia, two during exertion, with the final event (onset whilst cycling) requiring emergency direct current (DC) cardioversion for VT at an Accident and Emergency department. A repeat MRI at this stage confirmed normal systolic function, no valvular pathology and again an apical dyskinetic regional wall motion abnormality, with a non-enhancing filling defect. The patient, therefore, underwent ventricular aneurysmectomy via a standard median sternotomy, with complete excision of the LV apical aneurysm. A Cooley repair technique was performed, whereby the normal LV muscular edges were re-approximated using a running linear 4-0 Prolene suture in two layers with two reinforcing strips of Teflon felt (Figure 2). The operation involved a cardiopulmonary bypass (CPB) time of 22 minutes and an aortic cross-clamp time of 17 minutes. CPB was instituted using a double-stage venous cannula and a 21F arterial cannula. Cardiac arrest was achieved through the administration of intermittent cold-blood antegrade cardioplegia.

A post-operative trans-oesophageal echocardiogram (TOE) confirmed a good size residual LV cavity. The patient’s post-operative recovery was uneventful and he was discharged home on Day 6. He continued to make an excellent recovery, and an electrophysiology study three months later did not show a predisposition to any ventricular tachycardia.

## Discussion

Although LV apical aneurysms are not rare following MI, in this case, the patient had an unusual presentation in that he suffered complications of MI following a direct chest trauma despite the fact that his coronary angiogram was entirely normal on two occasions. We considered the first MI to be a result of the chest trauma and the second NSTEMI was likely a thromboembolic event from his LV aneurysm. The subsequent presentations 20 years later with episodes of ventricular tachycardia were a complication of the LV aneurysm. Ventricular arrhythmias associated with LVAs are believed to originate from an apical rim of scar tissue that acts as a zone of slow conduction [4]. Although an echocardiogram is a reliable tool for initial assessment, a cardiac MRI has the further advantage to provide information regarding detail on the size, wall structure of the LV aneurysm, and viability of the surrounding myocardium [5]. Trans-thoracic echocardiography (TTE) and TOE can still play an important role in diagnosis, especially intra-operatively when TOE provides valuable information such as LV and RV function [7]. Given the mechanism of arrhythmia, excision of the aneurysm with surrounding scar tissue is an effective treatment modality and usually resolves ventricular arrhythmias. In addition, the operation was offered in this case based on the presence of a large LV thrombus and the assumption that the aneurysm was the origin of the second (likely embolic) MI [3]. There are many surgical techniques that can be used for the repair of post-infarction LVA. These range from simple techniques, such as plication without opening the aneurysm, to more complex repairs, such as the Dor (endoventricular circular patch plasty) or McCarthy (endoventricular circular plasty without patch) techniques. In view of the relatively small size of this patient's LV apical aneurysm (2 cm), we utilised a “linear” or “sandwich” repair technique, which was first used by Cooley in 1958 and remains unchanged to this day [4]. Surgery for LVA remains a standard treatment with acceptable mortality, and favourable long-term results are seen for all of these techniques [3,8-9].

## Conclusions

Surgical treatment is indicated after the diagnosis of LV aneurysm to prevent future complications such as heart failure symptoms, risk of rupture, thromboembolic events or arrhythmias. Given this context, clinical suspicion should be raised by patients with a history of cardiac trauma and MI presenting with recurrent arrhythmias, and specialist referral considered if a thinned dyskinetic LV wall on echocardiogram or filling defect on MRI is visualised.
